# Familial Mediterranean Fever in Chinese Children: A Case Series

**DOI:** 10.3389/fped.2019.00483

**Published:** 2019-11-19

**Authors:** Ji Li, Wei Wang, Linqing Zhong, Junyan Pan, Zhongxun Yu, Shan Jian, Changyan Wang, Mingsheng Ma, Xiaoyan Tang, Lin Wang, Meiying Quan, Yu Zhang, Juan Xiao, Hongmei Song

**Affiliations:** Department of Pediatrics, Peking Union Medical College Hospital, Chinese Academy of Medical Sciences, Beijing, China

**Keywords:** Familial Mediterranean fever, MEFV protein, China, children, colchicine

## Abstract

**Background:** Familial Mediterranean fever (FMF) is an inherited auto-inflammatory disorder and is extremely rare in Chinese. This study aimed to investigate the demographic, clinical, and genetic features of FMF in a series of Chinese pediatric patients.

**Methods:** This was a retrospective case series of children with recurrent febrile or inflammatory episodes and referred to the Peking Union Medical College Hospital between 06/2013 and 06/2018. All suspected patients were genetically diagnosed and met the Tel-Hashomer criteria for FMF. Demographic, clinical, genetic, and treatment characteristics were collected. Descriptive statistics were used.

**Results:** Eleven patients were included (seven boys and four girls). The median age at the time of disease onset was 7.1 (range, 3–12) years, while the median age at diagnosis was 10.9 (range, 6–15) years. The median delay in diagnosis was 2.1 years (range, 6 months to 6.7 years). Fever (100%, 11/11) was the most common symptom, followed by joint pain (63.6%, 7/11), rash (54.5%, 6/11), abdominal pain (36.4%, 4/11), and oral ulcers (18.2%, 2/11), without evidence of amyloidosis. C-reactive protein (81.8%, 9/11) and erythrocyte sedimentation (90.9%, 10/11) were increased during attacks. All patients harbored one to five different MEFV mutations, with E148Q and L110P being the most frequent. A novel non-synonymous mutation F636Y in exon 10 was discovered. Favorable responses to colchicine was observed in all six treated patients.

**Conclusion:** The most common variants in our study were E148Q and L110P. F636Y may found for the first time. Colchicine led to favorable responses in all treated patients.

## Introduction

Familial Mediterranean fever (FMF) is the most common auto-inflammatory disorder with an autosomal recessive pattern of inheritance, predominantly affecting people of Mediterranean origin, such as Armenians, Arabs, Turks, and Jews (1/1,000–1/200) (1–4). Disease onset occurs at <10 years of age in the majority of patients (2–4).

Clinically, FMF is characterized by recurrent attacks of fever accompanied by serositis and/or arthritis at one or more sites, which manifests as abdominal pain, chest pain, or joint pain. Skin rash (i.e., erysipelas-like skin lesions) is frequently observed (2–4). These non-specific symptoms may be mistaken for other diseases, leading to misdiagnosis or delayed diagnosis, and even sometimes to unnecessary treatment and excessive operation (2, 5). Amyloidosis is the most severe complication of FMF, and can even be life-threatening if left untreated (2–4). Colchicine remains the primary choice of treatment in symptomatic FMF patients attributed to its key role in decreasing attack frequency as well as in preventing pathogenesis of amyloidosis (6, 7).

Almost all FMF cases are caused by point mutations (single substitutions) in the Mediterranean fever (MEFV) gene located on chromosome 16p13.3 (8). This gene encodes a protein of 781 amino acids called pyrin, which is mainly expressed in the myeloid lineage and is involved in the interleukin (IL)-1-associated inflammatory cascade (8, 9). Till now, 340 variants in the MEFV gene, including over 100 definitely disease-causing mutations (DCMs), have been documented in *Infevers* (https://infevers.umai-montpellier.fr/web/search.php?n=1) (8). The majority of variants are located in exons 2 and 10 (8), and some of them are also related to other serious, autoimmune, or auto-inflammatory diseases (10).

The prevalence of MEFV mutations and clinical symptoms of FMF varies greatly among different ethnic populations. Indeed, the prevalence in Turkey can be as high as 1 case per 150 persons, while the incidence can be as low as 2 cases per million persons in Eastern Europe (3). In China, FMF is recognized as being extremely rare, and the first cases of FMF in Chinese patients have been reported in 2018 (11). The current diagnostic criteria and therapeutic guidelines may not be optimally applicable to patients outside the Mediterranean region (12).

Therefore, the aim of the present retrospective study was to investigate the demographic, clinical, and genetic features of FMF in a series of 11 Chinese pediatric patients. This constitutes so far the largest cohort of pediatric Chinese patients with FMF. The results could provide clues for the diagnosis and management of FMF in the Chinese population.

## Materials and Methods

### Patients

In this retrospective case series, patients were recruited among those who were referred to the Department of Pediatrics of Peking Union Medical College Hospital from June 2013 to June 2018 for recurrent febrile or inflammatory episodes, which could not be completely attributed to infections, malignancy, or immunological diseases.

The inclusion criteria were: (1) age <18 years; (2) genetically confirmed homozygous or compound heterozygous for pathogenic MEFV variants; (3) clinically confirmed with FMF; and (4) the patients and their parents underwent genetic testing for MEFV mutations. The study was approved by the ethics committee of the Peking Union Medical College Hospital. The need for individual consent was waived by the committee.

### Genetic Detection

Whole exome next-generation sequencing was performed routinely in each patient and their parents. Sanger sequencing was performed at the detected mutation sites to exclude false-positive results and to verify the presence of co-segregation in all family members (2).

Candidate mutations were selected based on the following rationale: (1) detrimental, with a low frequency and reported to be pathogenic, with firm results (e.g., P115R); (2) reported to be pathogenic with controversial results (e.g., L110P, E148Q, E230K, G304R, P369S, and R408Q); and (3) detrimental, with a low frequency in the highly conserved region of exon 10 (e.g., M694V, V726A, M680I, and M694I). Those mutations once reported to be pathogenic but verified polymorphic later (such as R202Q) were excluded (13).

### Diagnostic Criteria and Therapy

FMF was diagnosed according to the conservative version of Tel-Hashomer criteria (Table 1) (14). A clinical diagnosis was made if the patient met one of the following requisites: (1) one major criterion; (2) two minor criteria; (3) one minor plus 5 supportive criteria; or (4) one minor plus at least four of the first five supportive criteria.

**Table 1:** Tel-Hashomer criteria for the diagnosis of FMF (14).

**MAJOR CRITERIA**
**Typical attacks**
1. Peritonitis (generalized)
2. Pleuritis (unilateral) or pericarditis
3. Monoarthritis (hip, knee, ankle)
4. Fever alone
**MINOR CRITERIA**
**1–3. Incomplete attacks**
1. Abdomen (localized, no signs of peritonitis)
2. Chest
3. Joint (arthritis not in hip, knee, ankle; ≥2 sites in the same attack)
4. Exertional leg pain
5. Favorable response to colchicine
**SUPPORTIVE CRITERIA**
1. Family history of FMF
2. Appropriate ethnic origin
3. Age <20 years at disease onset
**4–7. Features of attacks**
4. Severe, requiring bed rest
5. Spontaneous remission
6. Symptom-free interval
7. Transient inflammatory response, with one or more abnormal test result(s) for the white blood cell count, ESR, serum amyloid A, and/or fibrinogen
8. Episodic proteinuria/hematuria
9. Negative laparotomy or removal of a normal appendix
10. Consanguinity of parents

*Typical attacks are defined as recurrent (≥3 of the same type), febrile (rectal temperature of 38°C or higher), and short (lasting between 12 h and 3 days). Incomplete attacks are defined as painful and recurrent attacks that differ from typical attacks in one or two features: (1) the temperature is normal or lower than 38°C; (2) the attacks are longer or shorter than specified (but not shorter than 6 h or longer than a week); (3) no signs of peritonitis are recorded during the abdominal attacks; (4) the abdominal attacks are localized; and (5) the arthritis is in joints other than those specified*.

*FMF, Familial Mediterranean fever; ESR, erythrocyte sedimentation rate*.

All patients were treated according to the current management strategy for FMF (6). The treatment was tailored to each patient and included colchicine, methotrexate, prednisone, cyclosporine A, leflunomide, mycophenolate mofetil, hydroxychloroquine, and anti-infectious agents.

### Data Collection

Chinese ethnicity, sex, age at onset, age at diagnosis, diagnostic delay, previous history, family history, duration of fever, interval between fever episodes, abdominal pain, chest pain, joint symptoms, rash, other symptoms, response to colchicine, laboratory (erythrocyte sedimentation rate (ESR), C-reactive protein (CRP), IL-6, tumor necrosis factor (TNF)-α, Tel-Hashomer criteria, and gene mutations were extracted from the medical charts. All hematological tests were performed at the time of admission.

### Statistical Method

Data were managed using SPSS 16.0 (SPSS Inc., Chicago, IL, USA). Only descriptive statistics were used. Continuous variables are presented as median (range). Categorical data are presented as frequencies.

## Results

### Demographic Characteristics

A total of 11 Chinese pediatric patients harboring compound heterozygous or homozygous mutations and with clinical evidence of FMF were included (seven boys and four girls). The median age at the time of disease onset was 7.1 (range, 3–12) years, while the median age at diagnosis was 10.9 (range, 6–15) years. The median delay in diagnosis was 2.1 years (range, 6 months to 6.7 years). No patients had a positive family history of FMF or admitted parental consanguinity.

### Clinical Manifestations and Laboratory Findings

Fever (100%, 11/11) was the most common symptom, followed by joint pain (63.6%, 7/11), rash (54.5%, 6/11), abdominal pain (36.4%, 4/11), and oral ulcers (18.2%, 2/11). Other clinical manifestations included splenomegaly (9.1%, 1/11) and physical growth retardation (9.1%, 1/11) (Table 2). Amyloidosis was not observed. With regard to laboratory findings, CRP (81.8%, 9/11) and ESR (90.9%, 10/11) levels were increased in the majority of the patients during attacks. About 60% of patients (three of five tested patients) presented an increase in both serum IL-6 and TNF-α.

**Table 2:** Characteristics of the patients.

	**Patient 1**	**Patient 2**	**Patient 3**	**Patient 4**	**Patient 5**	**Patient 6**	**Patient 7**	**Patient 8**	**Patient 9**	**Patient 10**	**Patient 11**
**DEMOGRAPHIC CHARACTERISTICS**											
Nationality	Han	Han	Han	Han	Han	Han	Han	Han	Han	Han	Han
Sex	M	M	M	F	F	F	M	F	M	M	M
Age at onset	12 y 3 m	3 y 10 m	5 y 11 m	6 y 9 m	9 y 10 m	7 y 1 m	8 y 5 m	5 y 9 m	8 y	5 y 7 m	8 y 5 m
Age at diagnosis	12 y 9 m	13 y 5 m	6 y 7 m	9 y 3 m	10 y 11 m	12 y 2 m	15 y 1 m	6 y 8 m	11 y 3 m	7 y 8 m	9 y 2 m
Diagnosis delay	6 m	9 y 7 m	8 m	2 y 6 m	1 y 1 m	5 y 1 m	6 y 8 m	11 m	3 y 3 m	2 y 1 m	9 m
Previous history	Gastrointestinal perforation	Takayasu arteritis	–	–	–	–	–	–	Anaphylactoid purpura	Hydrocele of tunica vaginalis	–
Family history	–	–	–	–	–	–	–	–	–	–	–
**CLINICAL MANIFESTATIONS**											
Fever	Untypical	Untypical	Untypical	Untypical	Untypical	Untypical	Untypical	Typical	Typical	Untypical	Untypical
Duration	>1 week	>1 week	>1 week	<6 h	> weeks	> weeks	3 days	1 days	1 days	>1 week	>1 week
Interval	–	–	–	–	–	–	–	10–15 d	15–30 d	–	–
Abdominal pain	+ (severe)	+ (mild)		+ (mild)					+ (mild)		
Chest pain											
Joint symptoms	+			+	+	+	+			+	+
Rash					+	+	+		+	+	+
Other symptoms	Oral ulcer	Physical retardation	Splenome galy				Oral ulcer				
Response to colchicine		+	+	+		+			+		+
**LABORATORY FINDINGS**											
Inflammatory markers	ESR↑ CRP↑	ESR↑ CRP↑	ESR↑ CRP↑	ESR↑ CRP↑	–	ESR↑ CRP↑	ESR↑ CRP↑	ESR↑ CRP↑	ESR↑ CRP↑	ESR↑	ESR↑ CRP↑
Cytokines	–		IL-6↑ TNF-α↑			IL-6↑ TNF-α↑		–			IL-6↑ TNF-α↑
**TEL-HASHOMER CRITERIA**											
Major criteria	0	0	0	0	0	0	0	1	1	0	0
Minor criteria	2	1	1	2	1	1	1	0	1	1	2
Supportive criteria	2	5	5	3	5	5	5	2	2	5	3
**GENE MUTATIONS**											
Paternal	E148Q/P369S/R408Q/IVS8+8C>T		P369S/R408Q	P633L	E148Q	E148Q	L110P/E148Q	E230K	E148Q/P369S/R408Q/IVS8+8C>T	E148Q	E148Q
Maternal	G304R	E148Q	L110P/E148Q	E148Q/IVS8+8C>T	L110P/E148Q	E148Q	L110P/E148Q	P115R	E148Q	L110P/E148Q	E148Q
Novel		F636Y									

*ESR, erythrocyte sedimentation rate; CRP, C-reactive protein; IL-6, interleukin-6; TNF-α, tumor necrosis factor α; y, years; m, months*.

### Genetic Features

All of the included patients harbored different numbers (1–5) of MEFV variants. Six children were homozygotes for E148Q, and one of them was a homozygote for L110P. The other five patients had compound heterozygous mutations. A novel non-synonymous mutation F636Y in exon 10 was discovered. Ten distinct MEFV mutations were detected, respectively in exon 2 (L110P, c.329T>C; P115R, c.344C>G; E148Q, c.442G>C; E230K, c.688G>A; G304R, c.910G>A), exon 3 (P369S, c.1105C>T; R408Q, c.1223G>A), intron (IVS8+8C>T, c.1759+8C>T), and exon 10 (P633L, c.1898C>T; F636Y, c.1907T>A). E148Q (*n* = 16, 72.7%) was the most frequent, being heterozygous in four patients and homozygous in six patients, followed by L110P (*n* = 5, 22.7%), IVS8+8C>T, P369S and R408Q (*n* = 3, 13.6% each), P115R, E230K, G304R, P633L and F636Y (*n* = 1, 4.5% for each).

### Treatment and Prognosis

All patients received tailored management based on colchicine, methotrexate, prednisone, cyclosporine A, leflunomide, mycophenolate mofetil, hydroxychloroquine, and anti-infectious agents (Table 3). All six (100%) patients treated with colchicine after diagnosis experienced a significant decrease in the frequency and severity of attacks: two (33.3%) patients still had occasional fever attacks, while four (66.7%) were free of attacks. Three (50.0%) patients had normal inflammatory markers, and three (50.0%) patients had improved markers. The remaining five patients did not receive colchicine treatment due to various reasons including refusal by their families. Two (40.0%) of them still had occasional fever, one (20.0%) had a rash, and two (40.0%) were free from attacks. Three (60.0%) patients had normal inflammatory markers, and two (40.0%) patients had improved markers.

**Table 3:** Management of the patients with FMF.

	**Patient 1**	**Patient 2**	**Patient 3**	**Patient 4**	**Patient 5**	**Patient 6**	**Patient 7**	**Patient 8**	**Patient 9**	**Patient 10**	**Patient 11**
**TREATMENT**											
Colchicine	–	0.25 mg bid → 0.5 mg bid → 0.75–0.5 mg	0.25 mg qd → 0.5 mg qd → 0.75 mg qd	0.25 mg bid → 0.5–0.25 mg	–	0.25 mg qd → 0.5 mg qd → 0.5 mg bid	–	–	0.25 mg bid	–	0.25 mg bid → 0.25–0.5 mg → 0.5 mg bid
Others	MTX, prednisone	MTX, CsA, LEF, prednisone	–	CsA, prednisone	MMF, HCQ, LEF, prednisone	MTX, CsA, MMF, prednisone	Anti-infectious agent	Prednisone	Prednisone	MTX, HCQ, MMF, prednisone	MTX, prednisone
**OUTCOME**											
Clinical manifestations	Occasional fever	–	–	–	Occasional fever	Occasional fever	–	–	Occasional fever	Rash	–
Inflammatory markers	Improved	Improved	Improved	Normal	Improved	Improved	Normal	Normal	Normal	Normal	Normal
Last follow-up	Normal	Normal	Normal	Normal	Fever	Normal	Normal	Normal	Normal	Rash	Normal

*MTX, methotrexate; CsA, cyclosporine A; LEF, leflunomide; MMF, mycophenolate mofetil; HCQ, hydroxychloroquine*.

## Discussion

FMF has been well-described in Mediterranean populations (2–4). In terms of Asian ethnicity, hundreds of FMF cases had been reported in Japan (15, 16). A cohort of 11 adult FMF patients (11) was investigated in China. This study aimed to investigate the demographic, clinical, and genetic features of FMF in a series of Chinese pediatric patients. The results indicate that the most common variants in Chinese pediatric patients were E148Q and L110P. F636Y was found for the first time. Colchicine led to favorable responses in all treated patients. The present study, as far as we know, is the largest Chinese pediatric FMF cohort to date.

In the present case series, most patients experienced their first attack during their first decade of life. As an autosomal recessive disease, theoretically, FMF is considered to affect both genders equally, but previous studies indicated a slight female or male predominance (17, 18). No obvious gender predominance could be observed because of the small sample size of this study.

Several sets of criteria for clinical diagnosis have been established over the years, and the Yalcinkaya criteria (19), for example, are specific to children. Nonetheless, Chinese patients may not meet these criteria, as they are mostly based on the symptoms common in Mediterranean populations. Indeed, the new pediatric Turkish criteria did not improve FMF diagnosis compared with the Tel-Hashomer criteria in a mixed population of French children, even when using an appropriate control group, but the use of at least three Yalcinkaya's criteria can increase sensitivity (12, 20). In the present study, the criteria were chosen considering the low level of validation among non-Turkish populations and based on the popularity of the Tel-Hashomer criteria, which is the most widely used (2) and has been validated in adequate adult and pediatric cohorts. In our series, only two patients met one major criterion, while the others could not be clinically diagnosed without the introduction of supportive criteria, strongly suggesting marked differences in clinical manifestations between Chinese pediatric patients with FMF and Mediterranean FMF.

Though fever (up to a maximum temperature of over 38°C) was the most common symptom, most Chinese FMF children experienced incomplete attacks with long febrile durations and unfixed intervals. The typical fever episode lasts between 12 h and 3 days in Mediterranean patients. In the present study, only two children experienced a duration of 1 day while most of others fevered over 1 week once every attack. In addition, we cannot predict when the next episode will occur because of the variable intervals. Seven patients had joint symptoms, with only one with typical monoarthritis. More untypical sites like shoulder and wrist joints were involved symmetrically in six patients. Skin rash was the third most common symptom at various locations and in different patterns, mostly paralleling the course of fever. No erysipelas-like erythema has been observed in our cohort. Abdominal pain is the most frequent symptom in FMF in Mediterranean regions (21, 22), characterized by severe tenderness like an acute abdomen. In the present study, 36.4% of the Chinese FMF children had gastrointestinal symptoms, lower than in Mediterranean (90–98%), but similar to that in Japan (40–55%) (15, 16). In most patients, abdominal pain presented as a mild process unrelated to fever. On the contrary, 63.6% of adult FMF patients in China develop severe generalized abdominal pain with rebound tenderness (11). No obvious chest symptoms due to pleuritis (variable among Mediterranean FMF) have been observed in the present study. No clinical or laboratory evidence of amyloidosis was identified, indicating generally mild symptoms of Chinese FMF children. This finding is consistent with a previous study in Japan (23).

Attacks are associated with an increase in non-specific inflammatory mediators (24). In general, acute phase reactants like ESR and CRP would increase during attacks, while 1/3 of the cases maintain normal levels between episodes. In our cohort, ESR and CRP levels were elevated in 90.9% of patients. Increased levels of IL-6 and TNF-α were identified in 60% (3/5) of the tested patients. As inflammation markers, IL-6 and TNF-α have been used as therapeutic targets. Anti-TNF-α therapy and IL-6 inhibitors have demonstrated inspiring effects on FMF, especially for those with little response to colchicine (6, 25).

Differences in clinical manifestations may be partially explained by mutations in the MEFV gene. M694V, V726A, M680I, and M694I in exon 10, as well as E148Q in exon 2 are the most common “hot spots” for FMF, but these mutations, except E148Q, are not prevalent in Chinese children with FMF. Only two patients had mutations in exon 10 (F636Y and P633L), and among them, one had a detrimental pathogenic mutation with a low frequency (P115R). The remaining cases were compound heterozygotes (L110P, E148Q, E230K, G304R, P369S, and R408Q) with controversial results on pathogenicity. These mutations have been reported from symptomatic FMF, which are distributed in the normal population with a relatively high allele frequency of over 1%, even up to 30% (23, 26). In this study, 90.9% of patients had at least one mutation E148Q, which is more frequent than in patients with uncommon ethnic backgrounds like Japan (27). The E148Q mutation is related to a large spectrum of clinical manifestations, from mild symptoms (28) to severe complications (21). P369S and R408Q in exon 3 are always identified in cis, considered as polymorphic rather than definitively pathogenic mutations (29), and probably related to an atypical clinical presentation (30). In our cohort, three patients carrying P369S/R408Q presented with variable phenotypes: two experienced atypical attacks while the other one had typical fever. Similar observation has been obtained from L110P and E230K, which are more frequent in populations with low susceptibility to FMF and thus make much lesser contribution to clinical symptoms, especially when presenting together with a DCM from another exon (26). G304R tends to be benign. IVS8+8C>T was a hot spot in our patients. Though its impact on FMF has yet to be established, it provides a hint that disease-causing mutations may reside in intronic (non-coding) regions (20, 31).

We discovered a new missense mutation (F636Y) in exon 10 of the MEFV gene. One patient carried both F636Y and E148Q. His mother harbored one copy of E148Q while his father carried no mutation, suggesting this mutation was a *de novo* mutation. This patient suffered from atypical febrile attacks, mild abdominal pain, and Takayasu arteritis. According to the Tel-Hashomer criteria, he met one minor plus five supportive criteria and could be definitely diagnosed as FMF. We used the online bioinformatics tool PolyPhen-2 to predict the clinical significance of F636Y. This mutation was predicted as probably damaging (score: 0.998; sensitivity: 0.18; specificity: 0.98). It is the location rather than the type of mutation that determines the severity of FMF symptoms (26). Most mutations are located in exons 2 and 10. Mutations in exon 10, especially in the β30.2 domain, may produce a truncated protein pyrin, enhancing the interaction with caspase-1 and thus amplifying auto-inflammatory responses. No other mutations except exon 10 mutations were identified previously in individuals with severe FMF symptoms. We tend to consider F636Y as a pathogenic mutation, but we cannot confirm the cis or trans relationship between F636Y and E148Q in this patient because it is a novel mutation, and it is possible that the symptoms come from the E148Q mutation. More studies are guaranteed to validate this finding and to explore the role of F636Y in the pathogenesis of FMF.

The mode of autosomal recessive inheritance is widely accepted, but a study controversially describes that up to 30% of FMF patients possess only one of the well-known mutations, while about 25% present as compound heterozygotes (32). The only homozygous mutation observed in our cohort was E148Q or L110P. No obvious clinical differences were observed between homozygotes and compound heterozygotes. Importantly, symptomatic FMF patients may harbor none or only one of MEFV mutations, while carrying MEFV mutations may not always be accompanied by clinical symptoms. Besides the possibility of being polymorphic, a dosage-dependent effect may contribute to this genotype-phenotype dilemma. Variable expressivity, as well as incomplete penetrance, can also lead to this phenomenon (15, 21, 23). Given that the majority of patients carried more than two mutations, we speculate that these controversial variants were more likely to be conditionally pathogenic polymorphisms rather than definitely pathogenic mutations (26). Carrying more mutant loci may correlate with the onset of disease.

None of the patients had a positive family history in this cohort. The complexity of MEFV makes it difficult to perform genetic counseling. It should be noted that the diagnosis of FMF cannot rely on gene testing alone. Clinical diagnosis is the main approach, based on typical symptoms, family history, and response to colchicine treatment (23), after excluding other diseases with similar clinical features. If the clinical diagnosis is difficult to establish, gene testing can provide guidance for definitive diagnosis. Clinical diagnosis and genetic diagnosis are both needed. The strict inclusion criteria ensure the reliability of our cohort. As recommended by EULAR (6), colchicine should be used as early as possible after the diagnosis of FMF, and systematic evaluation made to formulate individualized treatment. A starting dose of <0.5 mg/day (<0.6 mg/day in case tablets contain 0.6 mg) is recommended for children <5 years of age, 0.5–1.0 mg/day (1.2 mg/day in case tablets contain 0.6 mg) for children 5–10 years of age, and 1.0–1.5 mg/day (1.8 mg/day in case tablets contain 0.6 mg) for children >10 years of age and for adults. The Tel-Hashomer criteria are partly based on colchicine responsiveness, and the introduction of colchicine in Chinese pediatric FMF patients has been reported before (33). Nevertheless, given the ethnic differences, we still need larger sample size to evaluate the efficacy of colchicine in Chinese patients. Six of the eleven patients received colchicine in our study, and all showed favorable responses. No obvious side effects were observed. Traditional anti-rheumatic treatments like glucocorticoids or immunosuppressants and new biological agents were also effective to different extents. There were no significant differences between the two groups receiving colchicine or not. Anti-TNF-α therapy and IL-6 inhibitors may help patients with resistance or unresponsiveness to colchicine, but our study did not involve these drugs.

This study has limitations. It is difficult to build up a direct relationship between genotype and clinical manifestations of Chinese FMF because of the small sample size and the even smaller numbers of patients with different mutations. In addition, there was no long-term follow-up. Because of the retrospective nature of the study, the data were limited to those available from the medical charts.

To the best of our knowledge, this study is by far the largest cohort of Chinese pediatric FMF, possibly highlighting several unique clinical and genetic characteristics in Chinese children with FMF. The findings could eventually help us modify and adapt to the diagnosis and treatment of FMF to Chinese pediatric patients. We expect more accurate, practical guidelines for the diagnosis and management of Chinese FMF in the near future.

## Data Availability Statement

The datasets used and/or analyzed in the present study are available from the corresponding author upon reasonable request.

## Ethics Statement

The studies involving human participants were reviewed and approved by the Peking Union Medical College Hospital. Written informed consent to participate in this study was provided by the participants' legal guardian/next of kin.

## Author Contributions

JL and WW designed the study and drafted the manuscript. ZY, SJ, CW, and MM delivered the intervention and completing data analysis. XT, LW, MQ, JX, and YZ performed the experiments. JL, WW, LZ, HS, and JP were the major contributors in writing the manuscript. All authors conducted data collection, read, critically reviewed and approved the final manuscript.

### Conflict of Interest

The authors declare that the research was conducted in the absence of any commercial or financial relationships that could be construed as a potential conflict of interest.
